# Hospital Festivals and Meetings

**Published:** 1895-04-13

**Authors:** 


### HOSPITAL FESTIVALS AND MEETINGS.

### BRISTOL ROYAL INFIRMARY.

The annual general meeting of the Governors of the Bristol Royal Infirmary was held on March 26th, Mr. C. D. Cave in the chair.

The President pointed out that a larger number of patients had altogether been treated during the past year than ever before in the history of the institution, though there had been a slight decrease in the number of in-patients, owing to the fact that the General Hospital was now in full working order, thus relieving some of the pressure. Two important improvements had been effected, in a much-needed increase in the out-patient accommodation, at a cost of about £1,300, and in the establishment of a laundry, with proper machinery, at a cost of £2,600. This was a large sum, but the committee felt that the saving in expense under the head of washing, and the great convenience of an efficient laundry on the premises, would be found to fully justify the outlay.

Other improvements effected, had been a new ward for obstetric patients, and certain repairs in the nursing home. He pleaded for continued and increased help towards carrying on the work of the infirmary. It was doing a great good in the city and neighbourhood, as was proved by the workmen's subscriptions. In conclusion, he thanked all those who had helped the cause of the charity, the honorary and resident staff, the matron and nurses. He was sorry to announce that Dr. Shekleton, their able and zealous secretary for so many years, was compelled, owing to failing health, to give up the post he had held so long. The report was unanimously adopted, and the usual votes of thanks closed the meeting.

### THE METROPOLITAN HOSPITAL.

The annual meeting of the governors of this hospital was held on Monday last in the board-room, Mr. Joseph Fry, J.P., chairman of the committee, in the chair. The report, which was duly adopted, pointed out the great need which existed for this hospital, situated as it is in the heart of a poor and densely crowded neighbourhood. The expenditure for the past year had, unfortunately, exceeded the receipts by some £1,346, but a gratifying increase in annual subscriptions had to be chronicled. There had been a considerable increase in the number of both in-patients and out-patients. The chairman, in acknowledging a vote of thanks accorded to him for presiding, said that he had taken a deep interest in the hospital for many years, but he felt that he could no longer tramp about asking for money as he used to. However, he still hoped to be of some service, and if only he could see the debt cleared, he would be satisfied. He trusted that the festival dinner would be a grand success, and that the committee would receive a very large sum on that occasion.

### PRINCESS MARY'S VILLAGE HOMES.

The yearly dinner in aid of the funds of these homes was held at the Hotel Metropole last week, the chair being taken by the Duke of Teck, in the absence of the Duke of York, who was not yet well enough to fulfil evening engagements. A goodly number of guests assembled, amongst whom were the Lord Chancellor, the Duke of Wellington, the Earls of Warwick, Yarborough, and Wharncliffe, Mr. Justice Hawkins, the Lord Mayor, and many others. The homes are intended for the care of little girls, the children of criminals, taken from the haunts of wickedness, to be brought up in the purer atmosphere of country homes. The children are divided into groups of ten, placed each in separate cottages under the care of some worthy woman, who acts as "mother" to the family. Here the ordinary English cottage life is carried on on the most strictly economical lines. There are some eighteen cottages in this little village, a school-house, infirmary, chapel, and laundry, about 190 children being there maintained in all. The Duke of Teck warmly commended the work being done, and asked for liberal contributions, stating that there was an annual deficit of £2,000. Donations were afterwards announced to the amount of £2,500. Of this £1,012 had been collected by the Duchess of York, and £1,020 by the Duchess of Teck.

